# Predicting and Preventing Epilepsy in Sturge-Weber Syndrome?

**DOI:** 10.15844/pedneurbriefs-30-11-3

**Published:** 2016-11

**Authors:** Csaba Juhász

**Affiliations:** 1 Department of Pediatrics and Neurology, Wayne State University School of Medicine, Children’s Hospital of Michigan, Detroit, MI

**Keywords:** Sturge-Weber syndrome, Epilepsy, Developmental Venous Anomaly

## Abstract

Investigators from the University of Montreal studied potential predictors of epilepsy in young patients with Sturge-Weber syndrome (SWS).

Investigators from the University of Montreal studied potential predictors of epilepsy in young patients with Sturge-Weber syndrome (SWS). They reviewed their database from 1990 to 2015 to identify SWS children followed in their institute. From 24 selected patients, 11 developed epilepsy and 13 did not after a mean follow-up of about 10 years. Patients with bilateral facial port-wine stain (PWS) had an increased risk for epilepsy (p=0.03), while location or extent of unilateral PWS was not associated with epilepsy risk. Interestingly, presence of developmental venous anomaly (DVA) on imaging was also a risk factor for epilepsy: this venous abnormality was present in 73% of epilepsy patients as compared to 25% of those with no epilepsy (p=0.03). Coincidence of bilateral PWS and DVA was associated with epilepsy in 100% of the cases. The study findings suggest that bilateral PWS and early detection of DVA on MRI both indicate a particularly high risk for epilepsy in SWS. Presence of these two features may guide preventive measures and/or tight follow-up in children with suspected SWS. [1]

COMMENTARY. Children with SWS are at high risk for seizures and neuro-cognitive deficits. However, the clinical course of SWS is highly variable, and early prognostic biomarkers of clinical outcome are still lacking. Facial PWS is often observed shortly after birth. Most of the PWS-affected children develop well, and only the minority of them will have intracranial involvement thus fulfilling the diagnostic criteria for SWS. However, conventional, contrast-enhanced T1-weighted MRI may not detect the leptomeningeal angiomatosis during the first year of life, which coincides with the common age of seizure onset in SWS. In some patients, however, DVAs may be visible on MRI before the occurrence of typical pial contrast enhancement. The study of Kaseka et al. [1] suggests that, in addition to bilateral PWS (a known risk factor for SWS-associated epilepsy), presence of such DVAs may be a prognostic imaging marker for future epilepsy in children suspected to have SWS. While the study was retrospective and included a limited number of subjects (n=24), this finding may have important clinical ramifications, if the results hold up in larger cohorts. Early, accurate prediction of imminent epilepsy may justify preventive treatment measures such as anti-epileptic therapy. A previous pilot study provided proof-of-principle data for the feasibility of such a preventive trial in SWS [2]. Recent developments in imaging techniques can improve sensitivity of detecting early SWS vascular abnormalities; for example, susceptibility-weighted imaging (SWI) was shown to be superior to conventional contrast-enhanced T1-weighted images for detecting SWS-associated DVAs [3]. Therefore, use of SWI and other advanced MRI techniques may enhance accurate detection of subtle DVAs during the early disease course. This could facilitate identification of high-risk SWS patients who may benefit from preventive medication. Whether such a treatment would have a disease-modifying effect, remains to be tested in prospective studies.

The pathomechanism of DVA development in SWS, and its role in epileptogenesis, are not fully understood. Kaseka et al. [1] discuss the possible association between SWS-associated DVAs and cortical dysplasia. Recent studies indeed provided evidence for previously unrecognized cortical malformations, mostly dysplasia in SWS [4]. Since cortical dysplasia is a highly epileptogenic pathology, DVA-associated dysplasia may increase the risk for epilepsy in SWS. However, this association, and its role in SWS-associated epilepsy, would require further confirmation from surgical specimens obtained from SWS epilepsy surgery in future studies.
